# Loneliness and mental health during the COVID-19 pandemic: A study among Dutch older adults

**DOI:** 10.1093/geronb/gbaa111

**Published:** 2020-08-05

**Authors:** Theo G van Tilburg, Stephanie Steinmetz, Elske Stolte, Henriëtte van der Roest, Daniel H de Vries

**Affiliations:** 1 Dept. of Sociology, Vrije Universiteit Amsterdam; 2 Dept. of Sociology, University of Amsterdam, and Institute for Social and Political Sciences, University of Lausanne, Switzerland; 4 Dept. on Aging, Netherlands Institute of Mental Health and Addiction, Trimbos Institute; 5 Dept. of Anthropology, University of Amsterdam

## Abstract

**Objectives:**

With the spread of COVID-19, the Netherlands implemented a policy to keep citizens physically distanced. We hypothesize that consequent reduction in the frequency of social contacts, personal losses and the experience of general threats in society reduced well-being.

**Methods:**

Data were collected from 1,679 Dutch community-dwelling participants aged 65 to 102 years old comprising a longitudinal online panel. Social and emotional loneliness and mental health were measured in May 2020, i.e., two months after the implementation of the measures, and earlier in October and November 2019.

**Results:**

In this pandemic, not only loneliness of older people increased, but mental health remained roughly stable. The policy measures for physical distancing did not cause much social isolation but personal losses, worries about the pandemic, and a decline in trust in societal institutions were associated with increased mental health problems and especially emotional loneliness.

**Discussion:**

The consequences of long-term social isolation and well-being must be closely monitored.

With the spread of COVID-19, the Netherlands implemented a policy to keep citizens physically distant from each other. While such measures are crucial to halt the spread of the infection, they might also have severe drawbacks for older adults who are often more socially isolated and lonely (Luhmann & Hawkley, 2016). These social risks are magnified if such measures remain in place for longer (United Nations, 2020). The Netherlands put in place an increasingly strict 'intelligent' lockdown from mid-March 2020. People were asked to stay at home, group activities prohibited, and many public facilities were closed. Visiting older people was explicitly discouraged and, for many, professional care was halted or limited. For more details about the Dutch case, please consult the Supplementary Material.

We expect that this pandemic has substantially increased loneliness and impaired mental health among Dutch older people. Perlman and Peplau (1981) defined loneliness as an unpleasant experience with a deficiency in a person's social relations. *Social loneliness* originates from the absence of a broader group of contacts, or an engaging social network. *Emotional loneliness* originates from the absence of an intimate figure or a close emotional attachment (Weiss, 1973). *Mental health problems* include being anxious, depressed and 'down, and not being peaceful and happy (Berwick et al., 1991). We take the absence of social and emotional loneliness, and unimpaired mental health together as well-being.

Hypothesizing about the consequences of this situation, we distinguish three causes that may reduce well-being. The first is reduction in the frequency of social contact. Most people have a strong need for social relationships in which they find solidarity, affection, and connectedness. This involves a close relative or friend with whom there is intensive contact, but also less intensive relationships, for example with someone from an organization with which there is infrequent superficial contact (de Jong Gierveld, van Tilburg, & Dykstra, 2018). Staying at home alone, or mainly with members of the household, for a longer period of time is an extreme disruption of social life. Decrease in the frequency of social contact leads to worsening of well-being (Hypothesis 1).

Second, people may experience personal losses because of the pandemic. Due to COVID-19 people may experience bereavement, losing a beloved spouse, or a person belonging to their social network. Feelings of loneliness and mental health problems are found to be the most serious consequences of bereavement (Perrig-Chiello, Spahni, Höpflinger, & Carr, 2016). Other types of loss include social contact, connections through a person's job or business, and participation in social and public activities. Furthermore, people may also lose the care and support provided by care or welfare professionals. Personal losses lower well-being (Hypothesis 2).

Third, the pandemic can be perceived at societal level as a source of stress that has psychological consequences. Reports on hospitalizations and severe pressure on intensive care facilities, as well as on the large increase in the number of deaths, particularly among older people, have dominated the media. General practitioners pro-actively discussed advance care planning with their vulnerable clients. Perceiving general threats in society contributes to a decline in well-being (Hypothesis 3).

Finally, lack of well-being urges people to cope with the situation. Lazarus and Folkman (1984) defined coping as the cognitive and behavioral ways in which individuals attempt to deal with specific demands of a situation that burden or exceed their resources. Active coping is behavior aimed at reducing the stress factor, in this case trying to maintain or increase social contact and connectedness despite the limitations due to the measures. Regulatory coping refers to reflecting on the stressor in order to reduce its effects, in this case reflecting on the situation causing the social constraints and general feelings due to the pandemic, to distract oneself from the negative situation. Both active and regulative coping increases well-being (Hypothesis 4).

## Method

*Respondents*

Data are collected from the Longitudinal Internet Studies for the Social Sciences (LISS) panel in the Netherlands. The random sample is drawn from the population register. Community-dwelling people in the sample were recruited by letter, followed by a telephone call or house visit (Scherpenzeel & Bethlehem, 2011). Respondents who were not able to participate digitally, were loaned equipment to provide access to the Internet and given training. Respondents are paid for each completed questionnaire. The overall assessment of the representativeness of the LISS probability-based panel was rather positive compared to online access panels (Knoef & de Vos, 2009). However, we cannot rule out a sample selection of older adults based on their health, capacity and willingness to use the Internet. For more information, see the Supplementary Material.

Between May 4 and May 26, 2020, 1,876 panel members aged 65 or older were asked to answer online questions on the social impact of physical distancing; 1,679 (90%) provided valid answers. Median completion time was thirteen minutes. The oldest was 102 years (M age = 73); 51% were male. Data on social-demographic characteristics were collected earlier. Comparative data on loneliness and mental health were collected in October and November 2019, respectively.

*Measuring instruments*

Social and emotional loneliness were both measured with three items (de Jong Gierveld & van Tilburg, 2010). Response options were ‘no,’ ‘more or less,’ and ‘yes.’ Scale scores were computed by counting the number of answers in the loneliness direction and ‘more or less’ and ranged 0-3. Reliability KR-20 was 0.77 and 0.62 in 2020, respectively for social and emotional loneliness, and 0.77 and 0.78 in 2018. For the five-item mental health inventory (Berwick et al., 1991) the six response options varied between (1) ‘never’ and (6) ‘ongoing.’ Answers on negative items were reversed. Cronbach’s alpha was 0.85 in 2020 and 0.87 in 2019. Scale scores were the mean of item scores.

Most of the independent variables were created ad hoc for this study. The survey questions and numerical values of the answers are provided in the Supplementary Material. Contact frequency was assessed by asking respondents how often they had contact in recent weeks and distinguishing ten categories of people, with response options ‘less than monthly or never’ to ‘(almost) daily.’ We also asked whether the contact frequency was different than before the pandemic, with response options ‘more,’ ‘equal,’ and ‘less.’

We asked whether the pandemic impacted respondents by referring to eleven situations. Response options were ‘no,’ ‘more or less,’ and ‘yes.’ We derived four scores: whether respondents were personally affected by own or other’s illness or by deaths; by social losses; by loss of work or activities; and by being outdoors less. We also asked whether they received or needed (professional) support on seven different domains, and assessed whether respondents had needs that were not met versus met.

Using data from Statistics Netherlands (2020) we computed excess mortality in the respondent’s municipality as the number of deaths in weeks 1-19 of 2020 minus the comparable number in 2019, per 10,000 citizens. We asked what the respondent’s estimate was of the likelihood of becoming ill due to the virus, how worried the respondent was about the pandemic, whether trust in four societal institutions (health care, science, government, Dutch society) had changed, and, with six questions, whether one followed governmental rules to avoid the proximity of others, or had been in quarantine.

For active coping, three items were on personal contact with others and five items were summarized as being involved in community-oriented actions. Three items were on regulative coping, e.g. finding distraction. Control variables are self-rated health, report of change in health in the last year, gender, age, living with a spouse or partner, the urbanity of the hometown, educational level and income.

*Procedure*

Change in loneliness and mental health between 2019 and 2020 was assessed by *t*-tests. We computed Cohen's (1988) *d* as effect size; 0.2 is a small, 0.5 a medium and 0.8 a large effect. Hypotheses were tested by estimating ordinal logistic regression models for loneliness and OLS models for mental health. Tolerance testing indicates the absence of multicollinearity between the independent variables; the lowest value was 0.66 for self-rated health. Missing values have been imputed (11% for the 2019 observations of loneliness; 10% for the 2019 observations of mental health; 6% for income; <1% for other variables); we created twenty data sets. The 2019 observation was included in the autocorrelation model. By adding variables in the subsequent four models the hypotheses were tested. Control variables were added in the final model.

## Results

Compared to seven months before, the respondents were more socially (Cohen's $d = 0.21$ indicates a small effect) and, especially, emotionally lonely ($d = 0.49$, a medium effect) during the pandemic (Table 1). For example, agreement with the item 'I miss having people around me'

increased by 34 percentage points from October 2019 to May 2020, and experiencing emptiness increased by sixteen percentage points. Compared with six months before, mental health improved significantly. However, the effect size was very small ($d$ = 0.17). Most change was observed for the item on depression, for which the answers ‘rarely’ or ‘never were given by 79% in November 2019 and by 83% in May 2020 (not shown in the table). The longitudinal correlation was the highest for mental health and the lowest for emotional loneliness. Social loneliness correlated 0.23 with emotional loneliness and -0.30 with mental health, and the latter two correlated -0.46.

Contact with children, children-in-law, and grandchildren was on average almost ‘at least weekly’ (Table 2). On average, contact frequency slightly decreased. Average contact frequency with other kin and non-kin was more than ‘at least weekly,’ and on average the respondents reported a decline. Few respondents were personally affected by their own or other’s illness or by deaths of relatives or friends. Many reported being personally affected by the loss of social contact, and fewer by loss of their own or someone else’s work or activities, or by being less frequently outdoors. Some respondents indicated that they had a personal, domestic, or social need that was not met. The spread of the virus and the number of sick and deceased varied widely across the Netherlands. Many municipalities in the south and east were badly hit, in contrast with most municipalities in the north that had almost no excess mortality (Statistics Netherlands, 2020). On average, respondents estimated their likelihood of being affected by the virus as similar to that of other people. They were not extremely worried about the pandemic. Trust in societal institutions increased. Almost everyone complied with physical distancing measures, and few were in quarantine. Coping was often active in terms of personal contact, but community-oriented coping was scarce. Many were involved in regulative coping.

The modelled variance in the three regression analyses of loneliness and mental health (Table 2) showed that Models 1 were scarcely an improvement over the autocorrelation models. The greatest improvement was found when variables on personal loss were added (Model 2), in particular for emotional loneliness. Investigating the general threats (Model 3) was of additional importance. The prediction in the models hardly improved when coping was added (Model 4).

Social loneliness increased among respondents who had little contact with children (-in-law) and grandchildren (Hypothesis 1), who were personally affected by being outdoors less, and who needed support but did not receive it (Hypothesis 2). Emotional loneliness increased among those who were personally affected by loss of social contact, work, and activities and by being less frequently outdoors, among those who needed support that was not received (Hypothesis 2), and among those with worries about the pandemic (Hypothesis 3). Mental health deteriorated when reporting the personal consequences of the pandemic, when being personally affected by being outdoors less and when needing support that was not received (Hypothesis 2), with worries about the pandemic, when trust in institutions declined, and when government rules were not followed (Hypothesis 3).

## Discussion

This research among the Dutch community-dwelling older adults in May 2020 shows that during the COVID-19 pandemic, average social loneliness increased slightly, and average emotional loneliness increased much more strongly. Comparison with the situation seven months earlier (October 2019) demonstrates the consequences of the pandemic.

To our knowledge there are only two other studies on loneliness before and during the pandemic. In cross-cohort analyses, Bu, Steptoe, and Fancourt (2020) assessed a loneliness prevalence of 37% among adults of all ages living in the United Kingdom in 2017-2019. During the pandemic, between March 21 and May 10, 2020, data were collected from adults recruited in different ways. In this sample they found a loneliness prevalence of 51%. The risk factors for loneliness were almost the same for the two samples but they did not specify their findings for respondents of different ages. Luchetti et al. (2020) collected data from adults living in the United States around the beginning of February 2020, the end of March and the end of April. There was no change in loneliness across the entire sample, but among respondents aged 65 years or older, loneliness increased slightly between February and March ($d$ = 0.14) and was stable thereafter. In response to the COVID-19 situation, resilience was shown by an increase in perceived support. In this context, our results corroborate the findings of increasing loneliness in these two studies, despite the differences in study design, location, and the context of national measures against the spread of the virus. However, we specifically observed a large increase in emotional loneliness. This might indicate that it was not so much the older adults' social embedding that was affected by the crisis, but rather the 'emptiness' and close connectedness with people around them.

The mental health of the Dutch older adults was roughly similar in May 2020 compared with six months earlier. A longitudinal Chinese study (Wang et al., 2020) shows a significant reduction in depression, anxiety, and stress four weeks after the COVID-19 outbreak, although levels of distress were still high. This may indicate that our data collection in May, two months after the start of the 'intelligent' lockdown in March, was too late to register the peak of the psychological impact. However, the outbreak in China (and much closer, in Italy) could also have prepared the Dutch mentally for the COVID-19 situation. Moreover, the number of deaths in many parts of the Netherlands was low compared to some regions, the capacity of intensive care after a major expansion was sufficient for every potential patient to be treated, and the Dutch lockdown was not very restrictive, so people had no problem with going outside. Many people may have seen that others, such as older people in nursing homes, were worse off. All of this may have contributed to the fact that, on average, mental health remained more or less stable compared with six months earlier.

Hypothesis 1 on the impact of reduced frequency of social contact on wellbeing did not find support. We observed that low contact frequency with children and grandchildren was related to increased social loneliness, but the reported change in contact frequency was not related. There may be several reasons for this lack of association. Six out of ten respondents lived with their spouse and therefore had frequent social contact. Many older adults may have sought alternative modes of contact and used contact via social media as a substitute for in-person contact (Quan-Haase, Mo, & Wellman, 2017). Physical distance measures may have (temporarily) lowered older adults' expectations of the frequency of contact and exchange in relationships, and, according to the cognitive discrepancy approach to loneliness (Rook & Peplau, 1982), this reduces their loneliness.

Hypothesis 2 on the experience of personal losses found support. Various personal losses and a non-fulfilled need for (professional) support were associated with increased social and emotional loneliness and mental health problems.

We found that various single factors of general threat were associated with decreased well-being, partly supporting Hypothesis 3. That emotional loneliness increased because of general perceived threat indicates that the concept is broader than just a lack of meaningful social relationships. The variation in municipal excess mortality was not related to a decrease in well-being, thus the locality did not matter.

From the literature it is known that coping by having personal contact is important in a crisis. Studying the response of Hong Kong Chinese people on the September 11, 2001 attacks on the United States and the SARS-outbreak in Hong Kong in 2003, Fung and Carstensen (2006)

showed that interaction with emotionally close social partners was beneficial. During the Iraqi SCUD missile attacks on Israel in 1991, North Israeli citizens had little control over the sources of stress. People did not evade reality, but applied active coping (Zeidner & Ben-Zur, 1993). However, based on our results we must reject Hypothesis 4. Our analyses showed that many applied active and regulative coping, but did not show that those who applied active or regulative coping had greater well-being during the COVID-19 pandemic than others. Unique to the COVID-19 crisis is today's broad access to communication technology that can help maintain social contacts remotely, and this may have been sufficient to allow people to cope with the pandemic. Access to the Internet is very high among Dutch people, including older adults, and in particular among participants in this study.

The pandemic affected many older people, but the community-dwelling people in this study were most likely much less severely affected than residents in nursing homes – not included in this study – who were completely cut off from visitors, and where fellow residents were dying (Gordon et al., 2020). A strong point of our study is that we were able to compare well-being in the pandemic with well-being at an earlier point in time, with a very low attrition. However, we have not included data on the occurrence of intervening 'normal' life events that may have caused the low modelled variance in change to well-being.

To conclude, in this pandemic, loneliness among community-dwelling older people increased two months after the implementation of measures for physical distancing, while mental health hardly changed. Social contacts are important for wellbeing, but we did not find many signs that frequency of social contact was important for understanding the change in well-being. Personal losses, concerns about the pandemic, and a decline in trust in societal institutions were associated with increasing mental health problems and especially emotional loneliness. These outcomes suggest that the most effective interventions necessary to combine physical distancing and wellbeing among older people living independently in the Netherlands are related to the *quality* of social contacts, including contact with societal institutions. Societal institutions could focus on facilitating meaningful social contacts with children and kin, ensuring support for needs including personal losses, and reducing the general perceived experience of a societal threat. While our results shed light on short-term effects after the start of the physical distancing measures, the consequences of long-term physical distancing on well-being need to be closely monitored.

## References

Berwick, D. M., Murphy, J. M., Goldman, P. A., Ware, J. E., Barsky, A. J., & Weinstein, M. C. (1991). Performance of a five-item mental health screening test. *Medical Care, 29*(2), 169-176.

Bu, F., Steptoe, A., & Fancourt, D. (2020). Who is lonely in lockdown? Cross-cohort analyses of predictors of loneliness before and during the COVID-19 pandemic. *medRxiv*. doi:10.1101/2020.05.14.20101360

Cohen, J. (1988). *Statistical power analysis for the behavioral sciences (second edition)*. Mahwah, NJ: Lawrence Erlbaum.

de Haas, M., Faber, R., & Hamersma, M. (2020). How COVID-19 and the Dutch 'intelligent lockdown' change activities, work and travel behaviour: Evidence from longitudinal data in the Netherlands. *Transportation Research Interdisciplinary Perspectives, 6*. doi:10.1016/j.trip.2020.100150

de Jong Gierveld, J., & van Tilburg, T. G. (2010). The De Jong Gierveld short scales for emotional and social loneliness: Tested on data from 7 countries in the UN generations and gender surveys. *European Journal of Ageing, 7*(2), 121-130. doi:10.1007/s10433-010-0144-6

de Jong Gierveld, J., van Tilburg, T. G., & Dykstra, P. A. (2018). New ways of theorizing and conducting research in the field of loneliness and social isolation. In A. L. Vangelisti & D. Perlman (Eds.), *The Cambridge handbook of personal relationships* (pp. 391-404). Cambridge: Cambridge University Press.

Fung, H. H., & Carstensen, L. L. (2006). Goals change when life's fragility is primed: Lessons learned from older adults, the September 11 attacks and SARS. *Social Cognition, 24*(3), 248-278. doi:10.1521/soco.2006.24.3.248

Gordon, A. L., Goodman, C., Achterberg, W., Barker, R. O., Burns, E., Hanratty, B., . . . Spilsbury, K. (2020). Commentary: COVID in care homes—challenges and dilemmas in healthcare delivery. *Age and Ageing*. doi:10.1093/ageing/afaa113

Knoef, M., & de Vos, K. (2009). *The representativeness of LISS, an online probability panel*. Retrieved from Tilburg, the Netherlands: https://www.lissdata.nl/sites/default/files/bestanden/paper_knoef_devos_website.pdf

Lazarus, R. S., & Folkman, S. (1984). *Stress, appraisal, and coping*. New York: Springer.

Luchetti, M., Lee, J. H., Aschwanden, D., Sesker, A., Strickhouser, J. E., Terracciano, A., & Sutin, A. R. (2020). The trajectory of loneliness in response to COVID-19. *American Psychologist*. doi:10.1037/amp0000690

Luhmann, M., & Hawkley, L. C. (2016). Age differences in loneliness from late adolescence to oldest old age. *Developmental Psychology, 52*(6), 943-959. doi:10.1037/dev0000117

Perlman, D., & Peplau, L. A. (1981). Toward a social psychology of loneliness. In R. Gilmour & S. W. Duck (Eds.), *Personal Relationships in disorder* (pp. 31-56). London: Academic.

Perrig-Chiello, P., Spahni, S., Höpflinger, F., & Carr, D. (2016). Cohort and gender differences in psychosocial adjustment to later-life widowhood. *The Journals of Gerontology, Series B, 71*(4), 765-774. doi:10.1093/geronb/gbv004

Quan-Haase, A., Mo, G. Y., & Wellman, B. (2017). Connected seniors: How older adults in East York exchange social support online and offline. *Information, Communication & Society, 20*(7), 967-983. doi:10.1080/1369118X.2017.1305428

Rook, K. S., & Peplau, L. A. (1982). Perspectives on helping the lonely. In L. A. Peplau & D. Perlman (Eds.), *Loneliness: A sourcebook of current theory, research and therapy* (pp. 351-378). New York: Wiley.

Scherpenzeel, A. C., & Bethlehem, J. G. (2011). How representative are online panels? Problems of coverage and selection and possible solutions. In M. Das, P. Ester, & L. Kaczmirek (Eds.), *Social and behavioral research and the Internet: Advances in applied methods and research strategies* (pp. 105-132). New York: Routledge.

Statistics Netherlands. (2020). Overledenen, provincie en gemeente, per week, 2020 [Deceased, province and municipality, per week, 2020]. Retrieved from https://www.cbs.nl/nl-nl/maatwerk/2020/24/overledenen-provincie-en-gemeente-per-week-2020

The National Institute for Public Health and the Environment. (2020). Ontwikkeling COVID-19 in grafieken [Development COVID-19 in graphics]. Retrieved from https://www.rivm.nl/coronavirus-covid-19/grafieken

United Nations. (2020). *The impact of COVID-19 on older persons*. Retrieved from https://unsdg.un.org/sites/default/files/2020-05/Policy-Brief-The-Impact-of-COVID-19-on-Older-Persons.pdf

Wang, C., Pan, R., Wan, X., Tan, Y., Xu, L., McIntyre, R. S., . . . Ho, C. (2020). A longitudinal study on the mental health of general population during the COVID-19 epidemic in China. *Brain, Behavior, and Immunity, 87,* 40-48. doi:10.1016/j.bbi.2020.04.028

Weiss, R. S. (1973). *Loneliness: The experience of emotional and social isolation*. Cambridge: MIT Press.

Zeidner, M., & Ben-Zur, H. (1993). Coping with a national crisis: The Israeli experience with the threat of missile attacks. *Personality and Individual Differences, 14*(1), 209-224. doi:10.1016/0191-8869(93)90191-5

Table 1. Loneliness and mental health in 2019 and 2020

| | 2019 | | 2020 | | | | | |
|---|---|---|---|---|---|---|---|---|
| | M | SD | M | SD | *r* | *t* | | Cohen's *d* |
| Social loneliness (0-3) | 0.95 | 1.15 | 1.17 | 1.20 | 0.62 | 8.2 | *** | 0.21 |
| There are plenty of people I can lean on when I have problems † | 0.26 | 0.44 | 0.33 | 0.47 | 0.46 | 6.0 | *** | 0.15 |
| There are many people I can trust completely † | 0.39 | 0.49 | 0.47 | 0.50 | 0.51 | 6.5 | *** | 0.17 |
| There are enough people I feel close to † | 0.26 | 0.44 | 0.36 | 0.48 | 0.39 | 7.8 | *** | 0.20 |
| Emotional loneliness (0-3) | 0.48 | 0.91 | 0.97 | 0.97 | 0.46 | 19.1 | *** | 0.49 |
| I experience a general sense of emptiness | 0.16 | 0.37 | 0.32 | 0.47 | 0.36 | 12.5 | *** | 0.32 |
| I miss having people around me | 0.21 | 0.41 | 0.55 | 0.50 | 0.27 | 23.8 | *** | 0.61 |
| I often feel rejected | 0.11 | 0.31 | 0.10 | 0.30 | 0.43 | -0.9 | | -0.02 |
| Mental health (1-6) | 4.93 | 0.75 | 5.02 | 0.73 | 0.71 | 6.4 | *** | 0.17 |
| Anxious † | 5.12 | 0.91 | 5.23 | 0.90 | 0.57 | 5.3 | *** | 0.14 |
| Down † | 5.50 | 0.80 | 5.62 | 0.73 | 0.51 | 6.1 | *** | 0.16 |
| Calm and peaceful | 4.51 | 1.01 | 4.56 | 1.02 | 0.51 | 2.2 | * | 0.06 |
| Depressed and gloomy † | 5.18 | 0.90 | 5.32 | 0.87 | 0.60 | 6.9 | *** | 0.18 |

| | | | | | | | | |
|---|---|---|---|---|---|---|---|---|
| Happy | 4.33 | 1.01 | 4.37 | 1.06 | 0.66 | 1.6 | | 0.04 |

Notes. N = 1,502 for the loneliness data. N = 1,521 for the mental health data. Scores are reversed for items marked with †. Loneliness items are dichotomous. Range for mental health items is 1-6.

* $p < .05$; ** $p < .01$; *** $p < .001$

Table 2. Regression of three aspects of well-being: Estimates from the final model (N = 1,697)

| Added in step | | M | SD | Social loneliness Estimate | | Emotional loneliness Estimate | | Mental health Beta | |
|---|---|---|---|---|---|---|---|---|---|
| | 2019 observation | | | 1.17 | *** | 0.96 | *** | 0.61 | *** |
| 1 | Contact frequency with children (-in-law), grandchildren (1-4) | 2.9 | 1.1 | -0.15 | ** | 0.00 | | 0.03 | |
| | Less frequent contact with children (-in-law), grandchildren (1-3) | 2.1 | 0.5 | -0.01 | | -0.05 | | -0.01 | |
| | Contact frequency with other kin, non-kin (1-4) | 3.2 | 0.8 | -0.07 | | -0.12 | | 0.02 | |
| | Less frequent contact with other kin, non-kin (1-3) | 2.3 | 0.4 | -0.06 | | -0.09 | | 0.00 | |
| 2 | Personally affected by own or other's illness, deaths (1-3) | 1.2 | 0.3 | -0.11 | | 0.29 | | -0.06 | *** |
| | Personally affected by loss of social contact (1-3) | 2.4 | 0.7 | 0.09 | | 0.42 | *** | -0.01 | |
| | Personally affected by loss of work, activities (1-3) | 1.7 | 0.4 | -0.18 | | 0.58 | *** | -0.02 | |
| | Personally affected by being outdoors less (1-3) | 1.6 | 0.6 | 0.22 | * | 0.42 | *** | -0.04 | * |
| | Support needed, but not received (vs. not needed or support received) | 0.08 | 0.27 | 0.65 | ** | 0.74 | *** | -0.06 | *** |
| 3 | Municipal excess mortality (per 10000) | 2.5 | 1.0 | 0.03 | | 0.05 | | 0.00 | |
| | Relative likelihood of becoming ill due to virus (1-5) | 2.8 | 0.9 | 0.08 | | 0.08 | | -0.01 | |
| | Worried about the pandemic (1-10) | 6.0 | 2.2 | 0.03 | | 0.10 | *** | -0.14 | *** |

| | | | | | | | | | |
|---|---|---|---|---|---|---|---|---|---|
| | Trust in societal institutions increased (1-5) | 3.5 | 0.7 | -0.14 | | 0.07 | | 0.05 | ** |
| | Following government rules: avoiding physical proximity (0-1) | 0.92 | 0.19 | -0.33 | | -0.20 | | 0.05 | ** |
| | Following government rules: quarantining (0-1) | 0.06 | 0.14 | -0.19 | | -0.13 | | 0.01 | |
| 4 | Active coping: personal contact with others (0-1) | 0.62 | 0.28 | -0.30 | | -0.09 | | 0.03 | |
| | Active coping: community-oriented (0-1) | 0.09 | 0.15 | -0.38 | | -0.29 | | 0.00 | |
| | Regulative coping: distraction, put into perspective (0-1) | 0.62 | 0.32 | -0.03 | | 0.24 | | -0.01 | |
| 5 | Self-rated health (1-5) | 3.0 | 0.8 | -0.02 | | -0.14 | | 0.05 | ** |
| | Health improved (1-5) | 2.9 | 0.6 | 0.05 | | -0.12 | | 0.08 | *** |
| | Female (vs. male) | 0.49 | 0.50 | -0.40 | *** | 0.26 | * | -0.06 | *** |
| | Age (65-102) | 72.9 | 6.0 | -0.02 | * | 0.01 | | 0.04 | * |
| | Living with spouse or partner (vs. not) | 0.61 | 0.49 | -0.34 | ** | -0.32 | ** | -0.01 | |
| | Urbanity of hometown (1-5) | 2.9 | 1.4 | 0.02 | | 0.04 | | -0.06 | ** |
| | Educational level (1-6) | 3.5 | 1.6 | 0.00 | | -0.03 | | -0.02 | |
| | Net household income €/1000 per month (0-15) | 2.8 | 1.5 | -0.04 | | 0.01 | | 0.00 | |
| Modelled variance ($R^2$) | | | | | | | | | |
| | Autocorrelation model | | | 0.39 | | 0.21 | | 0.50 | |
| | Model 1 | | | 0.40 | | 0.21 | | 0.51 | |

| | | | |
|---|---|---|---|
| Model 2 | 0.41 | 0.31 | 0.53 |
| Model 3 | 0.42 | 0.33 | 0.55 |
| Model 4 | 0.42 | 0.33 | 0.55 |
| Final model | 0.43 | 0.34 | 0.57 |

Notes. The range of independent variables refers to the numerical values of the answers in the Supplementary Material. Means and standard deviations are shown for non-imputed variables. The cut points and constant are not shown. In ordinal logistic regression, the link function is negative log-log. Estimates are from the final model of the pooled sample. In OLS regression, Beta is the standardized coefficient. $R^2$ is the mean across results from twenty imputed samples. From ordinal logistic regression, Nagelkerke $R^2$ is reported.

* $p$ < .05; ** $p$ < .01; *** $p$ < .001

.
