# Supplementary material for: Loneliness and Mental Health During the COVID-19 Pandemic: A Study Among Dutch Older Adults
Source: J Gerontol B Psychol Sci Soc Sci. 2020 Aug 5;76(7):e249–55. doi: 10.1093/geronb/gbaa111 (PMC7454922; doi:10.1093/geronb/gbaa111)
Supplement: gbaa111_suppl_Supplementary_Material [file gbaa111_suppl_supplementary_material.docx]

Supplementary Material

*Highlights of the COVID-19 crisis in the Netherlands*

The first reported case of COVID-19 in the Netherlands was on February 27, 2020. At the beginning of March, the number of confirmed cases started to grow rapidly, and on March 6 (in the mid of week of 10; Figure S1), the first death was reported. With the spread of COVID-19, the Netherlands implemented a policy to keep citizens physically at a distance.

On March 15, at the end of week 11 (Figure S1) and 17 days after the first confirmed cases, the ‘intelligent lockdown’ was put in place, meaning that people were asked to leave their homes only when necessary, i.e. for groceries, work that could not be done from home, or to get some fresh air or exercise. Everyone was asked to work from home, if possible. Restaurants, bars and ‘contact professions’ were closed, but people were free to decide when to leave their homes with up to two people and were asked to keep 1.5 meters from others. This was enforceable with a fine of 390€. People over 70 were advised not to have visitors. Changes in mobility were reported by de Haas, Faber, and Hamersma (2020). Approximately 80% of Dutch people reduced their activities outside the home, with a stronger decrease among older people. The number of trips and distances covered decreased by 55% and 68% respectively compared to the fall of 2019, but walking and cycling gained in popularity.

At the beginning of April, the number of new infections was still rising but stabilized by the end of April. A staged easing of the ‘intelligent lockdown’ was announced on May 6, starting with the resumption of most ‘contact professions’ on May 11, with primary schools slowly reopening from mid-May onwards and official advice changing from ‘only leave the house when necessary’ to ‘stay home as much as possible’ on June 1.

Figure S1. Development of the COVID-19 crisis in the Netherlands

Note: The number of deceased, the incidence of patients in the hospital and the incidence of all patients are COVID-19 related. The excess mortality is calculated as the number of all deaths in 2020 minus the comparable number in the same week in 2019 per 1,000,000 inhabitants. Data collection started in week 19 and was completed on the second day of week 22, with the median day of response on May 11, i.e., the first day of week 20.

Sources: The National Institute for Public Health and the Environment (2020); Statistics Netherlands (2020)

*The LISS panel*

The LISS panel is a household panel of the Dutch (speaking) population aged 16 years and older that started in 2007 and is administered by CentERdata. The recruitment of the panel members is based on a random sample of addresses drawn from the community registers in close co-operation with Statistics Netherlands. All selected households were contacted in a traditional way, either by telephone or in person (<https://www.lissdata.nl/about-panel/sample-and-recruitment>). If there was no computer and/or Internet connection in the household, CentERdata provided a so-called ‘simPC,’ which is especially developed for older people having no experience with computers (with large buttons and a screen designed to be easily readable). The final panel membership rate was 48% of the total gross sample.

In the first year of recruitment, older adults were under-represented (Knoef & de Vos, 2009). Due to several stratified refreshment samples, this underrepresentation could be reversed. LISS carried out three refreshment samples to compensate for attrition and to correct for sample bias by oversampling the difficult to reach groups that had a below-average response in the main recruitment. The first refreshment sample was added in 2009, and was stratified by age, ethnicity, and household type. The second refreshment sample was added in 2011-2012 and was based on the original sample design. The third and fourth refreshment samples were added in 2013-2014 and 2016-2017 and were stratified. From the latest available report from 2015, it is evident that the refreshment has even led to a small overrepresentation of the 65+: 23% in LISS in comparison with 21% in the population (<https://www.lissdata.nl/sites/default/files/bestanden/Representativiteit%20van%20het%20LISS%20panel%202015.pdf>).

*Description of the questionnaire*

The survey and the data will become publicly available in English and Dutch at <https://www.dataarchive.lissdata.nl/>. Variables created ad hoc for this study were based on the following questions.

Contact frequency

We would like to ask you a few questions about contact with people who do not live in your house. By contact we mean going on a visit (they come to visit you or you go to visit them), making a phone call, writing to them, emailing them, WhatsApp, contact via computer or video calls, having a chat with them.

- Children living away from home
- Grandchildren living away from home
- Daughters and sons-in-law
- Other family
- Friends and acquaintances
- Neighbors
- Shop assistants

Questions for each category:

- How often have you been in contact with these people in recent weeks?
  Response options: (1) less often or never; (2) approximately monthly; (3) at least weekly; (4) (almost) every day; (-) not applicable
- Is the contact now, in Corona time, different from before?
  Response options: (1) more; (2) not changed; (3) less; (-) not applicable

Personal losses

In this Corona era, everyday life has changed a lot. Through this crisis, are there situations and events that affect you personally?

*Personally affected by own or other’s illness*

- I have been sick
- The death or serious illness of your partner or a housemate
- The death or serious illness of a family member, friend, or close acquaintance

*Personally affected by loss of social contact*

- No, less or other contact with grandchildren and children
- No, less or other contact with other family, friends, and acquaintances
- Discontinuation of normal leisure activities, such as club activities
- Can no longer visit cafes, restaurants, and many shops

*Personally affected by loss of work, activities*

- Loss of your job, business and financial problems of your own
- Loss of work, business and financial problems of your partner, family member, friend, or close acquaintance

*Personally affected by being outdoors less*

- Fewer opportunities to exercise outdoors, such as walking, cycling, and sports
- Necessary groceries (such as food, medication) are harder to get

Response options: (1) no; (2) more or less; (3) yes

Have you received help or support with the following activities in the past two weeks?

- Maintaining social contacts (e.g. via computer or tablet/iPad)
- Daily activities
- Financial situation (debts, administration, making payments)
- Mental health (e.g. meaning, coping with anxiety, gloominess)
- Physical health and dealing with medication
- Personal care (e.g. washing, dressing, support stockings)
- Housekeeping (e.g. cooking, shopping, cleaning)

Response options: yes, support from the same person/organization as before Corona time; yes, support from someone else or another organization; yes, both from the same person/organization as before Corona time; no, but I do need support; no, support is not needed. We assessed whether respondents had a need of support in one or more domains that was not met, versus all other alternatives.

General threats

If you compare yourself to other people, how much chance do you have of getting sick from the Corona virus?

Response options: (1) a much smaller chance; (2) a smaller chance; (3) an equal chance; (4) a bigger chance; (5) a much bigger chance

How worried have you been about the Corona crisis during the last seven days?

Response options: 1 stands for ‘I don’t worry’ and 10 for ‘I am extremely worried’

Has the way in which the Corona crisis is being tackled in the Netherlands changed your confidence in the following four institutions?

- The healthcare sector
- Science
- The government
- Dutch society

Response options: (1) decreased sharply; (2) decreased; (3) not changed; (4) increased; (5) strongly increased; (-) do not know. The average score across the items was computed.

Which of the following pieces of advice have you followed in the past week?

*Following government rules: avoiding physical proximity*

- Do not go to crowded places
- Avoid public spaces
- Keeping a distance from others (1.5 meters)

*Following government rules: quarantining*

- Adjusting regulations for school or work
- Going into quarantine because you have symptoms
- Going into quarantine even if you have no symptoms

Response options: tick what applies. The count of the affirmative answers was rearranged to 0-1.

What are you doing, or have you done, to break through your social isolation or loneliness in the Corona era or to deal with the situation? In some activities we give examples.

*Active coping: personal contact with others*

- Contact with others through technology (emailing, (video) calling, texting, talking via social media, sharing videos and photos)
- Contact at 1.5 meters distance (talking to neighbors, walking, or visiting at a distance)
- Joint activities with housemates (baking, games, gardening)

*Active coping: community oriented*

- Participation in playful neighborhood actions (balcony music, street bingo, window concert)
- Participation in actions for others (clapping, burning candles)
- Group meetings via technology (virtual neighborhood walk, online group meeting, or TV daytime activities)
- Seeking spirituality (praying, meditating, reading spiritual texts)
- Volunteering or helping acquaintances

*Regulative coping: distraction, put into perspective*

- Relativizing (humor, think that I am well off compared to some others, think that we can handle this problem in the Netherlands)
- Finding distractions in and around the house (gardening, hobbies, TV, computer games)
- Outdoor distraction (hiking, biking, sports, driving a car, going to shops)

Response options: tick what applies. The count of the affirmative answers was rearranged to 0-1.

**Reference**

de Haas, M., Faber, R., & Hamersma, M. (2020). How COVID-19 and the Dutch “intelligent lockdown” change activities, work and travel behaviour: Evidence from longitudinal data in the Netherlands. *Transportation Research Interdisciplinary Perspectives*, 6, 100150. doi:10.1016/j.trip.2020.100150
